# Efficacy of ALK inhibitors on NSCLC brain metastases: A systematic review and pooled analysis of 21 studies

**DOI:** 10.1371/journal.pone.0201425

**Published:** 2018-07-27

**Authors:** Fausto Petrelli, Chiara Lazzari, Raffaele Ardito, Karen Borgonovo, Alessandra Bulotta, Barbara Conti, Mary Cabiddu, Jody Filippo Capitanio, Matteo Brighenti, Mara Ghilardi, Luca Gianni, Sandro Barni, Vanesa Gregorc

**Affiliations:** 1 Oncology Unit, Oncology Department, ASST Bergamo Ovest, Treviglio (BG), Italy; 2 Oncology Unit, Department of Oncology, Division of Experimental Medicine, IRCCS San Raffaele, Milan, Italy; 3 Oncology Unit, IRCCS Centro di Riferimento Oncologico della Basilicata (CROB), Via Padre Pio 1, Rionero in Vulture (PZ), Italy; 4 Surgical Oncology Unit, Surgery Department, ASST Bergamo Ovest, Treviglio (BG), Italy; 5 Neurosurgery Unit, Department of Neurosurgery and Gamma Knife Unit, IRCCS San Raffaele Scientific Institute, Milan, Italy; 6 Oncology Unit, Oncology Department, ASST Ospedale di Cremona, Viale Concordia 1, Cremona, Italy; University of Nebraska Medical Center, UNITED STATES

## Abstract

**Background:**

Patients with anaplastic lymphoma kinase rearranged (ALK+) non-small cell lung cancer (NSCLC) have a higher risk of developing brain metastases (BMs) than patients with other NSCLC sub-types. ALK inhibitors have activity in BMs due to ALK+ NSCLC. We performed a systematic review of the literature with the aim of assessing the efficacy of ALK inhibitors on BMs.

**Material and methods:**

A systematic search of the literature was performed using the databases Pubmed, EMBASE, Web of Science, The Cochrane Library, and SCOPUS. Relevant publications reporting activity of ALK inhibitors in NSCLC BMs were retrieved. Data were pooled using the number of events/number of evaluable patients according to fixed or random effect models. Intracranial tumour response was assessed through overall response rate (ORR), disease control rate (DCR: ORR + stable disease rate), median progression-free survival (PFS), and overall survival (OS). The primary endpoint was intracranial overall response rate (IC ORR).

**Results:**

A total of 1,016 patients with BMs from 21 studies were analysed. In patients receiving ALK inhibitors in the first line setting, the pooled IC ORR was 39.17% (95%CI 13.1–65.2%), while the pooled IC ORR observed in further lines was 44.2% (95%CI 33.3–55.1%). Intracranial disease control rate (IC DCR) was 70.3% and 78.2% in naïve and pre-treated patients, respectively. Patients who had not received brain radiation attained an IC ORR of 49.0%.

**Conclusions:**

Based on these data, ALK inhibitors are effective in both naive and pre-treated patients with similar IC ORR and IC DCR, irrespective of the line of therapy.

## Introduction

During the last ten years, the technological advances and the deeper knowledge of non-small cell lung cancer (NSCLC) biology have revolutionized the management of patients with NSCLC. The discovery of activating mutations in the epidermal growth factor receptor gene (EGFR) [1], and the identification of the gene rearrangement between echinoderm microtubule-associated protein like 4 and anaplastic lymphoma kinase (EML4-ALK) [2], have initiated the era of precision medicine in lung oncology, thus significantly improving survival in molecularly classified subsets of patients, who are amenable to targeted inhibition.

EML4-ALK translocations are observed in approximately 5% of NSCLC patients, manly never or light smokers, with a median age of 52 years and adenocarcinoma histology [3]. ALK positive NSCLC patients have a high risk of developing brain metastases (BMs), as observed in at least 20% of cases at the time of the initial diagnosis, thus dramatically influencing patients’ quality of life and their prognosis [4]. Local therapies (surgical resection, stereotactic radio surgery, and whole brain radiotherapy) are generally used for the management of patients with BMs, since the central nervous system (CNS) is considered a pharmacological sanctuary, where the expression of drug-efflux transporters limits the blood-brain barrier penetration. The concomitant use of systemic tyrosine kinase inhibitors (TKIs) and local treatments prolong patients’ survival, as observed in a retrospective analysis, including 90 ALK positive NSCLC patients who reached a median overall survival (OS) of more than four years [5]. A double median survival was observed in TKI naive patients compared with those who developed BMs during treatment with ALK inhibitors. Ceritinib, alectinib, brigatinib, and lorlatinib have been designed to overcome the pharmacodynamic and pharmacokinetic crizotinib failure at brain site.

In the current paper, we performed a pooled analysis, including data from ALK positive NSCLC patients with BMs receiving ALK inhibitors. Patients were stratified according to the type of ALK inhibitors, the line of treatment, and if they had previously received radiotherapy or not. The intracranial activity of the different ALK Inhibitors and their influence on intracranial progression free survival (IC PFS) and OS was evaluated, as the effect of radiotherapy on intracranial objective response rate (IC ORR).

## Methods

### Search strategy and selection criteria

We have systematically searched PubMed (MEDLINE), EMBASE, The Cochrane Library, Scopus, and Web of Science for relevant prospective studies published between inception and 30^th^ June 2017. The following keywords were used: *alk [All Fields] AND ("lung neoplasms” [MeSH Terms]) OR ("lung"[All Fields] AND “neoplasms" [All Fields]) OR "lung neoplasms” [All Fields] OR ("lung"[All Fields] AND “cancer" [All Fields]) OR "lung cancer” [All Fields] OR ("carcinoma*, *non-small-cell lung" [MeSH Terms] OR ("carcinoma" [All Fields] AND "non-small-cell" [All Fields] AND “lung" [All Fields]) OR "non-small-cell lung carcinoma” [All Fields] OR “nsclc" [All Fields] AND ("brain metastases” [All Fields] OR "central nervous system metastases” [All Fields])*. Preferred reporting items for systematic reviews and meta-analyses (PRISMA) guidelines were followed when planning, conducting, and reporting this meta-analysis (S1 Table).

The studies included had to satisfy the following criteria: (1) randomised control trials (RCTs), or prospective or observational studies; (2) ≥ 10 patients included; (3) enrollment of ALK positive NSCLC patients with BMs; (4) treatment with an ALK inhibitor. Case reports and series where the concomitant use of radiotherapy was permitted were excluded. Our search included journal articles written in English and non-English. Two reviewers independently determined study eligibility (FP and RA). Disagreements were resolved by consensus with a third author (CL).

### Statistical analysis

For each study included in the meta-analysis, we computed the type of study, the total number of patients treated, the frequency of patients with BMs, the type of ALK inhibitor used, the use of local treatments, the ICC ORR, the IC PFS, the median progression free survival (PFS), the median OS, and the one-year OS. The primary endpoint was ICC ORR. We pooled data on ICC ORR and intracranial disease control rate (ICC DCR) that reflected the proportion of patients with complete response, partial response, or stable disease for at least 24 weeks. We employed the random-effect model as a conservative approach to account for different sources of variation among studies (i.e. within-study variance and between-study variance) [6].

Secondary endpoints were IC DCR, median PFS, median OS, and one-year OS. Dagra software was used to ascertain survival data by digitising figures if the information was not provided directly. Finally, we computed both Q and I^2^ statistics in order to evaluate heterogeneity across studies. A significant Q value indicates the lack of homogeneity of results across studies. I^2^ estimates the proportion of observed variance that reflects real differences in effect sizes.

First, we performed a subgroup analysis according to the line of therapy (first line vs. beyond). For each subgroup, a further analysis including race (Asiatic vs. others), previous radiotherapy (yes vs. no), and type of ALK inhibitor was performed. To check the stability of our findings, sensitivity analyses were conducted. We computed the ICC ORR changes by removing one study at a time.

Quality of trials was assessed by the Jadad scale for randomized controlled studies and using the Newcastle-Ottawa Scale (NOS) for retrospective cohort studies. The risk of bias for the studies included deemed to be eligible for the review was assessed independently by two review authors (FP and CL) using the Cochrane ‘Risk of bias’ assessment tool. Discrepancies were resolved by discussion. Trials were screened and analysed for the following risk of bias criteria: 1. Selection bias (random sequence generation and allocation concealment); 2. Performance bias (blinding of participants and personnel); 3. Detection bias (blinding of outcome assessment); 4. Attrition bias (incomplete outcome data); 5. Reporting bias (selective reporting), and 6. Other bias. To determine whether published studies had different results from unpublished studies, publication bias analyses using the Egger’s regression method and the Begg and Mazumdar’s rank correlation test were performed. In both tests, the absence of publication bias was indicated by non-significant results. Descriptive statistics (pooled mean and meta-analysis of proportions) were calculated with Comprehensive Meta-Analysis software, version 3 (Biostat, Inc.).

## Results

**Fig 1:**
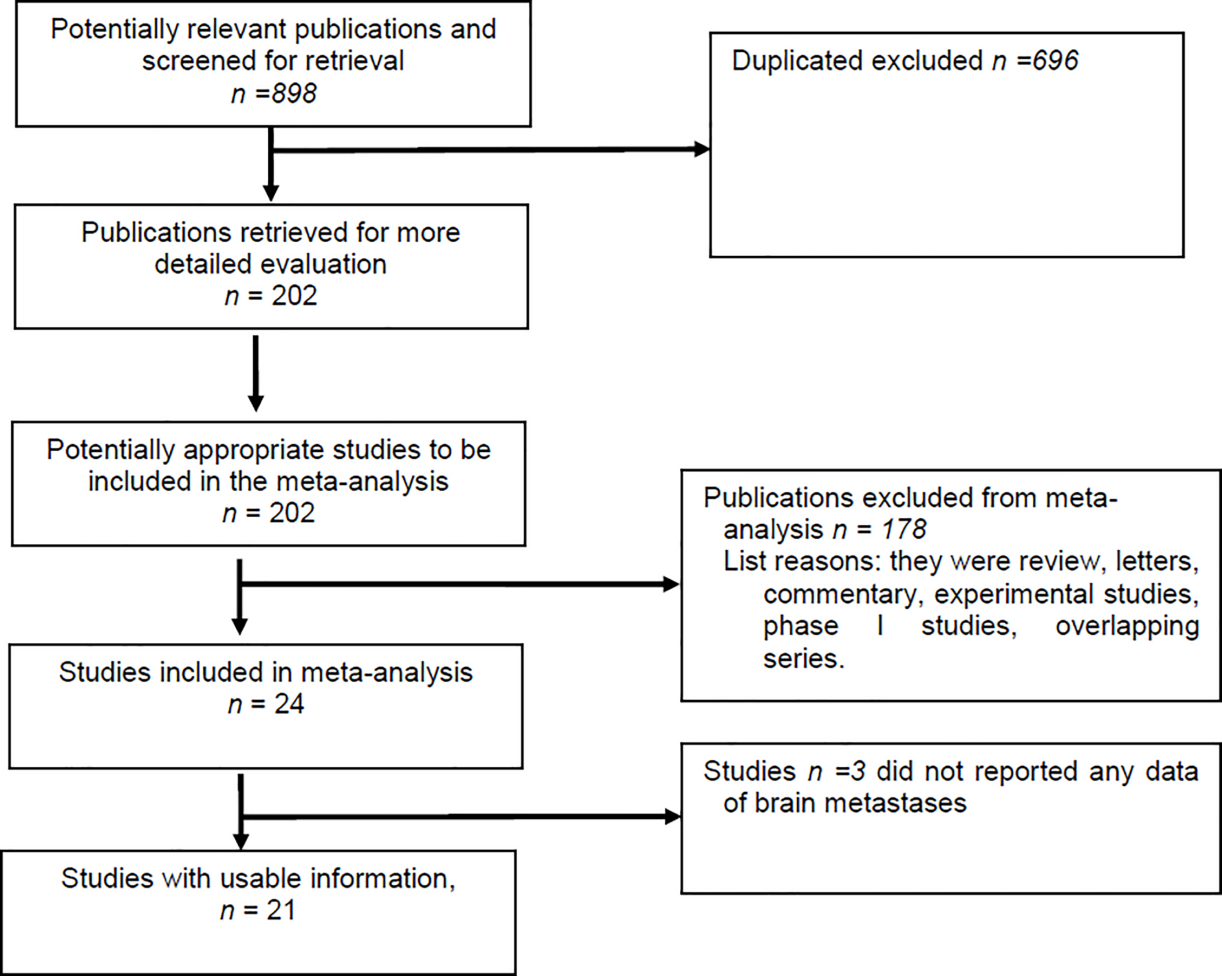
Overview of trials search and selection.

Eight hundred and ninety eight publications were retrieved (Fig 1) and 21, which included data from 1,016 patients with ALK positive NSCLC and BMs, were analysed (range 7–275) (Table 1). Seven studies evaluated crizotinib [7–13], five ceritinib [14–18], four alectinib [19–22], one both crizotinib and alectinib [23], one included different ALK inhibitors [5], and two evaluated brigatinib [24, 25]. In one series, the ALK inhibitor(s) used were not specified [26]. Four studies were conducted in the first line setting [9, 18, 23, 26], 14 studies included patients pre-treated with ≥ one line of therapy [5, 7, 8, 12–17, 19–22, 25], and three a cohort of patients receiving ALK inhibitors in different lines (first or beyond) [10, 11, 24].

**Table 1:** Characteristics of included studies.

Author/year	N° of pts	Type of study	Country	ALK inhibitor	Previous local therapy %	Previous ALK inhibitor %	Previous CT %	IC ORR %	ORR criteria
**Bendaly 2017**	22	Retrospective	USA	Ceritinib	-	100	20.7	70	Recist ^§^
**Costa 2015**	22	Phase 2/3	International	Crizotinib	0	0	100	18	Recist 1.1
**Costa 2015**	18	Phase 2/3	International	Crizotinib	100	0	100	33	Recist 1.1
**Crino 2016**	25	Phase 2	International	Ceritinib	100	100	100	45	-
**Davis 2017**	23	Retrospective	Europe	Crizotinib	52	69^	-	31.2**	-
**Doherty 2017**	21	Retrospective	Canada	Crizotinib/Ceritinib	95	0	0	-	-
**Gadgeel 2014**	21	Phase 1/2	US	Alectinib	81	100	95	52	Recist 1.1
**Gadgeel 2016**	41	Phase 2	International	Alectinib	0	100	80	58.5	Recist 1.1
**Gadgeel 2016**	95	Phase 2	International	Alectinib	100	100	80	35.8	Recist 1.1
Gettinger 2016*	21	Phase ½	USA/Spain	Brigatinib	0	85	-	57.1	Recist 1.1
**Gettinger 2016**	46	Phase ½	USA/Spain	Brigatinib	100	91	-	41.3	Recist 1.1
**Hong 2017**	15	Retrospective	China	Crizotinib	66.7	69.7^	-	-	Recist 1.1
**Johung 2010**	90	Retrospective	US	Crizotinib (93%)	93	-	-	-	Recist 1.1
**Kim 2016**	94	Phase 1	International	Ceritinib	67	80	-	23.4	Recist 1.1
**Kim 2017**	44°	Phase 2	International	Brigatinib	-	65	74	52.2	Recist 1.1
**Lei 2015**	19	Retrospective	China	Crizotinib	0	63.2	-	73.7	Recist 1.1
**Lei 2015**	19	Retrospective	China	Crizotinib	100	63.2	-.	63.2	Recist 1.1
**Metro 2016**	7	Retrospective	Italy	Alectinib	73	100	-	85.7	Recist 1.1
**Peters 2017**	58	Phase 3	International	Crizotinib	37.9	0	0	26	Recist 1.1
**Peters 2017**	64	Phase 3	International	Alectinib	42.1	0	0	59	Recist 1.1
**Shaw 2017**	66	Phase 3	International	Ceritinib	56	100	100	35°°	Recist 1.1
**Solomon 2016**	39	Phase 3	International	Crizotinib	100	0	0	-	Recist 1.1
**Soria 2017**	54	Phase 3	International	Ceritinib	100	0	0	46.3	Recist 1.1
Soria 2017*	32	Phase 3	International	Ceritinib	0	0	0	46.9	Recist 1.1
**Tamura 2017**	14	Phase 1/2	Japan	Alectinib	-	0	100	-	Recist 1.1
**Xing 2016**	20	Retrospective	China	Crizotinib	60	74	-	15	-
**Yoshida 2016**	26	Retrospective	Japan	Crizotinib	50	69^	-	20^^	Recist 1.1

*, subgroup analysis with no previous radiotherapy patients of the main study

°, only patients with measurable brain metastases

^, previous therapies not specified

§, version not specified

IC ORR, overall response rate of brain metastases

**, only for second/later lines patients

°°, in 17 patients with measurable disease

^^, in 10 patients with brain metastases present before crizotinib; CT, chemotherapy; US, United States of America

-, not reported.

### Intracranial overall response rate and disease control rate

**Fig 2:**
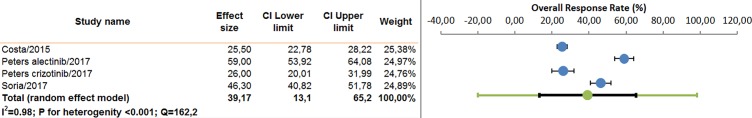
Pooled analysis of intracranial overall response rate (first line trials).

In patients receiving ALK inhibitors in the first line setting, data on IC ORR and IC DCR were available in three out of five studies analyzed. The pooled ICC ORR was 39.17% (95%CI 13.1–65.2%), and the pooled IC DCR was 70.3% (95%CI 47.7–86.0%), according to the random effect model (Fig 2). The ICC ORR observed in patients receiving alectinib was 59.0% (95%CI 29.3–83.0%), in those treated with ceritinib was 56.6% (95%CI 33.3–77.4%), and in those receiving crizotinib was 26.0% (95%CI 8.9–55.9%). Analysis by race was not possible, as the majority of the trials analyzed were international multicentre or US studies.

**Fig 3:**
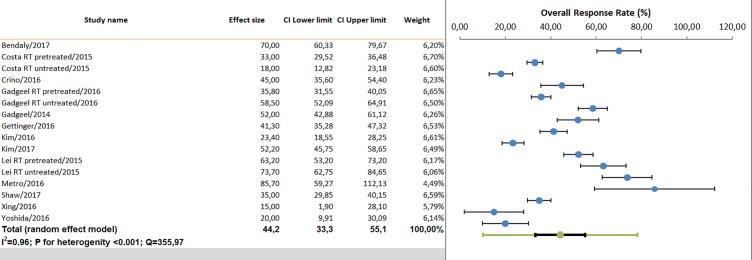
Pooled analysis of intracranial overall response rate (second line or beyond trials).

In the pre-treated setting, 12 studies evaluated IC ORR and nine IC DCR. The pooled IC ORR observed was 44% (95%CI 33.3.-55.1%), and the IC pooled DCR was 79% (95%CI 70–85.9%), according to the random effect model (Fig 3). The IC ORR observed in patients receiving alectinib was 52.4% (95%CI 34.1–70.1%), in those treated with brigatinib was 46.7% (95%CI 25.2–69.5%), in the group under ceritinib was 41.5% (95%CI 28–56%), and in those under crizotinib was 35.3% (95%CI 17.9–57.8%). The efficacy of ALK inhibitors was not dependent by previous radiotherapy, as observed in meta-regression analysis (p = 0.64). Comparable results were found in Asiatic vs. western/international populations.

In an exploratory analysis, including five studies enrolling patients who had not previously received brain radiotherapy, the IC ORR was 49.0% (95%CI 27.3–71.1%). The pooled IC ORR, including data from patients who had undergone radiotherapy (range 14–100 of pre-treatment with radiotherapy) was 38.4% (95%CI 31.1–46.3%). However, meta-regression analysis did not find any significant difference in terms of IC ORR between patients who had previously received radiotherapy or not (p = 0.32).

Overall, IC DCR was 85.8% (95%CI 79.3–90.4%), 88.3% (95%CI 80.5–93.2%), 69.1% (95%CI 61.8–75.6%) and 71.2% (95%CI 51.7–85.1%) for alectinib, brigatinib, ceritinib and crizotinib, respectively.

### Intracranial progression free survival, median progression free survival, median overall survival, and one-year overall survival

Median PFS in naive patients was 7.3 months (range 5.9–10.7), and median IC PFS was 13.2 months (range 7.0–15.7). Median OS was 23 months. Pooled one-year OS was calculated using data from two studies, and was 64.0% (range 59.0–81.0%).

In the pre-treated setting, median PFS was 8.0 months (range 4.4–38.0), and median IC PFS of 14.6 months (range 8.0–22.3). Median OS, available in two studies, was 23.0 months. Pooled one-year OS, using data from four studies, and was 71.4% (range 31.0–76.2%).

### Publication bias

Quality of trials and risk of bias according to the Cochrane ‘Risk of bias’ are reported in Table 2. No evidence of bias was observed (Begg’s funnel plot and Egger’s test were not significant) when including data from the studies enrolling patients in the first line setting or in more advanced lines (S1 and S2 Figs). After the one-study-removed procedure, the results of pooled IC ORR did not change, thus confirming that no dominant study was included.

**Table 2:** Assessment of the risk of bias and quality assessment of studies included in the meta-analysis.

AUTHOR/YEAR	TYPE OF STUDY		RISK OF BIAS					QUALITY OF STUDIES
		Selection bias	Performance bias	Detection bias	Attrition bias	Reporting bias	Other bias	JADAD/ NOS
**Bendaly 2017**	Retrospective	NA	NA	Low	Low	Low	Low	-/7
**Costa 2015**	Phase 2/3	Moderate	Moderate	Low	Low	Low	Low	3/-
**Costa 2015**	Phase 2/3	Moderate	Moderate	Low	Low	Low	Low	3/-
**Crino 2016**	Phase 2	NA	NA	NA	Low	Low	Low	NA
**Davis 2017**	Retrospective	NA	NA	NA	Unclear	Unclear	Low	-/6
**Doherty 2017**	Retrospective	NA	NA	NA	Low	Low	Low	-/6
**Gadgeel 2014**	Phase 1/2	NA	NA	NA	Low	Low	Low	NA
**Gadgeel 2016**	Phase 2	NA	NA	NA	Low	Low	Low	NA
**Gadgeel 2016**	Phase 2	NA	NA	NA	Low	Low	Low	NA
Gettinger 2016*	Phase 1/2	NA	NA	NA	Low	Low	Low	NA
**Gettinger 2016**	Phase 1/2	NA	NA	NA	Low	Low	Low	NA
**Hong 2017**	Retrospective	NA	NA	NA	Low	Unclear	Unclear	-/7
**Johung 2016**	Retrospective	NA	NA	NA	Low	Unclear	Unclear	-/8
**Kim 2016**	Phase 1	NA	NA	NA	Low	Low	Low	NA
**Kim 2017**	Phase 2	NA	NA	NA	Low	Low	Low	NA
**Lei 2015**	Retrospective	NA	NA	NA	Low	Low	Low	-/6
**Lei 2015**	Retrospective	NA	NA	NA	Low	Low	Low	-/6
**Metro 2016**	Retrospective	NA	NA	NA	Low	Low	Unclear	-/7
**Peters 2017**	Phase 3	Moderate	Moderate	Low	Low	Low	Low	4/-
**Peters 2017**	Phase 3	Moderate	Moderate	Low	Low	Low	Low	4/-
**Shaw 2017**	Phase 3	Moderate	Moderate	Low	Low	Low	Low	4/-
**Solomon 2016**	Phase 3	Moderate	Moderate	Low	Low	Low	Low	4/-
**Soria 2017**	Phase 3	Moderate	Moderate	Low	Low	Low	Low	4/-
Soria 2017*	Phase 3	Moderate	Moderate	Low	Low	Low	Low	4/-
**Tamura 2017**	Phase 1/2	NA	NA	NA	Low	Low	Low	NA
**Xing 2016**	Retrospective	NA	NA	NA	Low	Low	Low	-/7
**Yoshida 2016**	Retrospective	NA	NA	NA	Low	Low	Unclear	-/7

*, subgroup analysis with no previous radiotherapy patients of the main study

NOS, Nottingham Ottawa Scale; NA, not applicable

## Discussion

Patients with ALK positive NSCLC are generally young and have a substantial risk of developing BMs. With the introduction of ALK inhibitors and the OS increase obtained with these agents, IC control has become more important. In the current paper, we performed a pooled analysis that included data from ALK positive NSCLC patients with BMs receiving treatment with ALK inhibitors, who had been pre-treated or not with radiotherapy and/or chemotherapy. Our results indicate that ALK inhibitors are effective at the brain site. Comparable IC ORR was observed in patients receiving ALK inhibitors in first or further lines. This efficacy was not dependent by previous radiation.

According to the current guidelines, the treatment strategy suggested for ALK positive patients who develop BMs at the time of diagnosis is systemic therapy with an ALK-inhibitor.Local therapy (radiotherapy or surgery) might be eventually delayed at the onset of symptoms for intracranial disease.

Despite crizotinib activity at brain site, with an IC ORR of 18%, the appearance of BMs is observed in 60% of cases during the course of crizotinib [7]. Crizotinib pharmacokinetic failure is mainly due to its poor blood-brain barrier penetration [27], as it is a substrate of P-glycoprotein and human ATP-binding cassette subfamily efflux transporters.

For these reasons, in the presence of asymptomatic crizotinib brain failure, local treatment and the continuation of crizotinib is generally an accepted strategy, despite no prospective clinical trial has compared this option with the shift to second or third-generation ALK inhibitors at the time of brain progression. In the case of symptomatic multifocal brain progression, local therapy and the shifting to another ALK inhibitor is the preferred choice.

An unanswered clinical question is whether withhold brain radiation in patients receiving second and third generation ALK inhibitors, since these agents penetrate the blood-brain barrier better than crizotinib and offer a significant control of brain disease. This represents a significant challenge, especially considering that whole brain radiotherapy might decrease cognitive function and reduce memory in a young subgroup of patients carrying ALK rearrangement who have a high probability of extended survival (up to four years) after the diagnosis of BMs, as has been recently observed [5]. Further prospective studies are needed to prove that the omission of brain radiotherapy does not negatively impact survival. Only a small fraction of patients (20%-40%) entered into the clinical trials and included in the current meta-analysis were not treated with radiation because in the majority of the studies, the presence of unstable brain lesions was an exclusion criterion. This might have influenced the results of our analysis. Moreover, the activity of ALK inhibitors in the brain was a secondary end point of the trials evaluated, and patients were not stratified according to the number of brain lesions, previous radiation therapy, and the type of radiotherapy used. Furthermore, we have to consider that only a small percentage of the patients had measurable brain lesions (approximately 30%) at the study enrollment. Results from the prospective ongoing NCT02521051 trial, which aims to evaluate the role of alectinib and bevacizumab, and the ASCEND-7 (NCT02336451) trial, designed to assess the efficacy of ceritinib in ALK-positive NSCLC patients with BMs or leptomeningitis who are progressing on crizotinib and who are not treated with radiotherapy, will help to better define the optimal sequence of brain radiation and systemic ALK inhibition.

Our results confirm improved IC control with ceritinib, alectinib, and brigatinib compared with crizotinib. Half the patients included in the ASCEND-1 trial had asymptomatic or controlled BMs [15]. Of the 74 patients evaluable, only 34% had measurable lesions. Comparable results were observed in the phase II ASCEND-2 trial that enrolled 140 NSCLC patients who were progressing on crizotinib [14]. Approximately 70% of the enrolled patients had brain lesions at study entry, 70% of whom had received radiotherapy before starting to use ceritinib. Despite ceritinib being transported by the human ATP-binding cassette sub-family, its binding is stronger to hABCG2 than hABCB1 [28], and a higher expression of hABCB1 has been found in the human blood-brain barrier. This suggests that hABCG2 might have a stronger influence on the accumulation of ceritinib in the brain compared to hABCB1, and indicates that an adequate drug concentration might be obtained. Alectinib is not a substrate of P-glycoprotein, and effective therapeutic concentrations have been measured in the CSF of patients (2.69 nmol/L, which is above the IC50 concentration needed for ALK inhibition) [19]. Brain tumor regression was observed in mice receiving alectinib and in ALK-positive NSCLC patients not previously treated with brain radiotherapy [19, 20, 22]. There is also evidence that alectinib has activity in patients with leptomeningeal carcinomatosis [29]. A significant reduction in tumor burden in the brain was observed in mice treated with brigatinib compared with those under crizotinib [30]. The efficacy of brigatinib in the CNS was also confirmed in phase I and II clinical trials [24, 25]. According to the available data, the dose of brigatinib influences the IC ORR, with higher efficacy in those patients receiving 180 mg daily compared to those treated with 90 mg daily.

This is the first pooled-analysis evaluating the efficacy of ALK TKIs in NSCLC patients with BMs using the largest series of data available. Our analysis has several limitations. First, we have not evaluated individual patient data, but we have extracted information from published papers. Second, the quality of the data was heterogeneous, and several relevant types of information, such as the number of BMs, the status of extracranial disease control, the use of salvage therapies, and the health-related quality of life outcomes were not consistently reported. Third, the data refer to both first and further lines of therapy.

Despite the limits discussed above, our results suggest that withholding immediate brain radiotherapy in patients with asymptomatic/oligometastatic BMs and use radiotherapy in case of progression could be a valid option, even though further prospective trials are needed to confirm the efficacy of this strategy. Alectinib is more effective than crizotinib in terms of IC PFS, IC ORR, and overall PFS, and it should be considered as the preferred choice, as confirmed by the ALEX and J-ALEX trials [23, 31].

In conclusion, there is evidence, albeit of limited quality, that ALK positive NSCLC patients with BMs derive significant clinical benefit from ALK inhibitors with or without previous (whole) brain radiotherapy, and the efficacy is similar to that observed for extracranial systemic disease.

## Supporting information

S1 Table PRISMA 2009 checklist. (DOC)

S1 Fig Funnel plot for first line studies response rate analysis. (DOCX)

S2 Fig Funnel plot for second line studies response rate analysis. (DOCX)
