# Supplementary figures and images for: Efficacy of ALK inhibitors on NSCLC brain metastases: A systematic review and pooled analysis of 21 studies
Source: PLoS One. 2018 Jul 27;13(7):e0201425. doi: 10.1371/journal.pone.0201425 (PMC6063430; doi:10.1371/journal.pone.0201425)

**
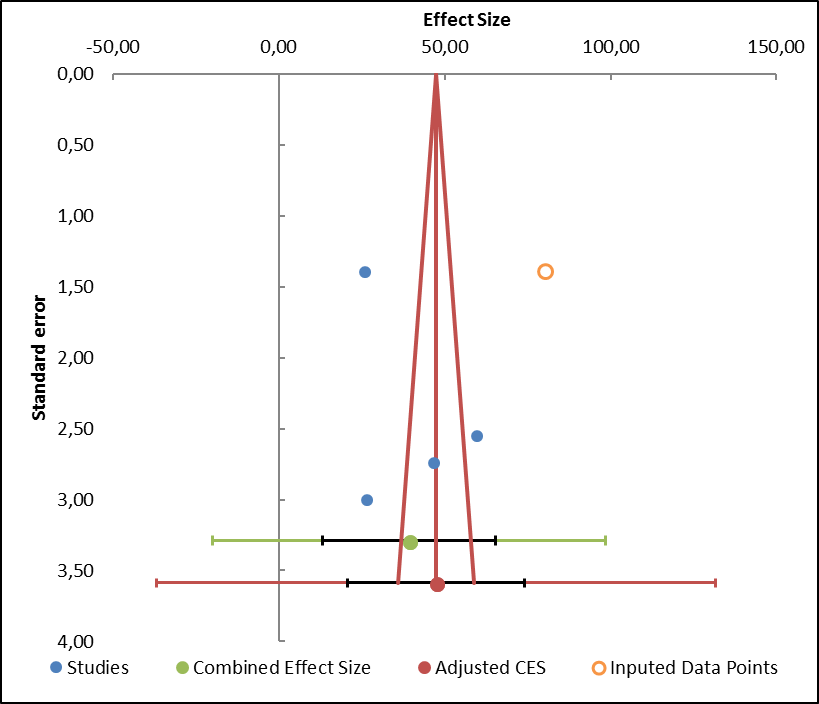
**

**Supplemental Fig.1 Funnel plot for first line studies response rate analysis**

Supplement: S1 Fig — (DOCX) [file pone.0201425.s002.docx]

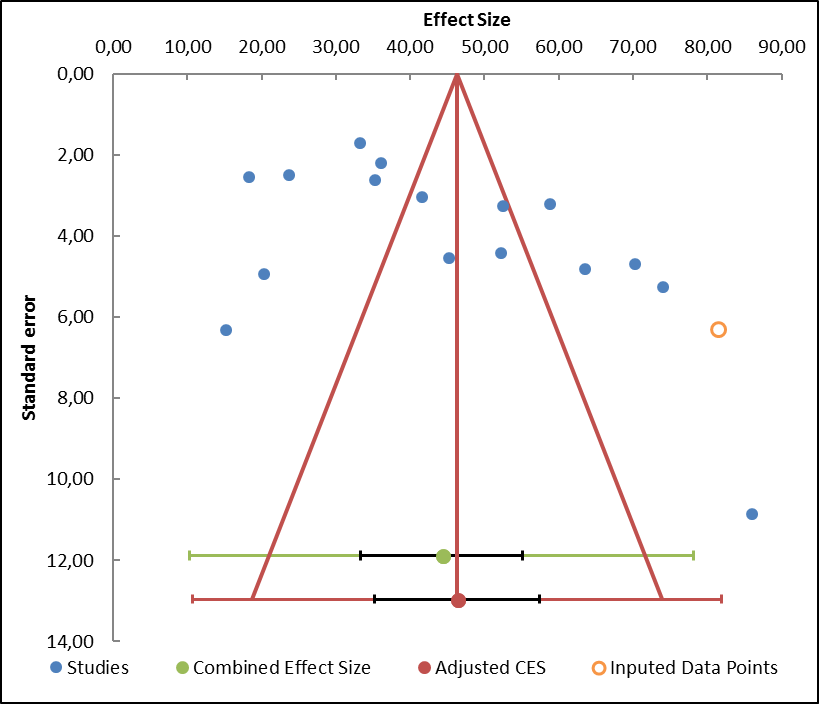


**Supplemental Fig.2 Funnel plot for second line studies response rate analysis**

Supplement: S2 Fig — (DOCX) [file pone.0201425.s003.docx]
